# Paradox mindset as an equalizer: A moderated mediated perspective on workplace ostracism

**DOI:** 10.1371/journal.pone.0294163

**Published:** 2024-02-07

**Authors:** Alina Ahmad, Sharjeel Saleem, Rizwan Shabbir, Beenish Qamar

**Affiliations:** 1 Lyallpur Business School, Government College University Faisalabad, Faisalabad, Pakistan; 2 Faisalabad Business School, National Textile University, Faisalabad, Pakistan; Guangxi Normal University, CHINA

## Abstract

Incorporating the conservation of resources theory as a comprehensive framework, this study investigates a cohesive conceptual model analyzing the impact of workplace ostracism on employees’ innovative work behavior (IWB). The investigation further delves into the mediating influence of perceived control and the moderating roles of paradox mindset and support for innovation. Data collection employed a survey approach involving three-time lags through questionnaires administered to 513 employees within Pakistan’s public sector organizations. The hypothesized relationships were evaluated using conditional process modeling. Our research sheds light on how perceived control mitigates the negative impact of ostracism. The paradox mindset is identified as a key moderator influencing cognitive resources and navigating ostracism. Support for innovation enhances the link between perceived control and innovative work behavior. In addition, limitations, future research directions, and implications of our findings for fostering creative workplaces are also discussed.

## Introduction

In today’s rapidly evolving business landscape, organizations are increasingly recognizing the critical role of innovation in maintaining a competitive edge and achieving sustained success [1]. Innovation not only fuels product development and market expansion but also promotes organizational growth and adaptability. Thus, organizations find themselves leaning heavily on their employees’ capacity for innovative work behavior (IWB) to meet escalating demands and navigate the ever-shifting market conditions [2, 3]. Innovative work behavior refers to intentionally creating and implementing novel ideas within a work role in an organization to enhance and benefit the performance of a job role, group, or organization [1]. It provides an opportunity to create and implement original conceptions, ultimately improving organizational performance [1]. Thus, in this context of fostering innovative work behavior (IWB) as a cornerstone of organizational success, it becomes imperative to delve into the workplace dynamics that can either hinder or promote IWB.

One often overlooked yet potent factor is workplace ostracism [4, 5]. This subtle yet impactful phenomenon entails the discreet exclusion, isolation, and neglect of individuals by their colleagues or superiors [6]. This phenomenon, characterized by behaviors like ignoring, exclusion, and withholding information, carries far-reaching negative consequences for employees’ well-being, job satisfaction, and overall performance. It has been revealed by extant research that ostracism leads to numerous deleterious outcomes, including burnout [7], emotional exhaustion [8], unsafe behaviors [9], and a terroristic mindset [10], among others. Among all other forms of workplace mistreatment, workplace ostracism is more detrimental to the well-being of employees [11]. Such negative experiences undermine psychological and emotional well-being [12, 13].

Furthermore, in contrast to the prevailing notion that workplace ostracism might be an infrequent phenomenon, emerging research reveals a distinct and pervasive reality. A survey conducted by a Chinese recruitment platform showed that more than 70% of respondents were victims of workplace ostracism [14]. Moreover, another survey conducted in 262 companies based in America showed that 66% of the employees claimed that they were systematically excluded by their colleagues and have experienced ostracism. These experiences result in the feeling of invisibility, and employees start questioning their worth at their workplace. Thus, ostracism negatively affects an individual’s self-perceptions, such as self-esteem and identity [15].

While the detrimental influence of workplace ostracism is well-documented, its potential impact on an individual’s ability to engage in IWB remains an area that warrants deeper investigation [2, 3]. Thus, scholars have called for research that investigates the intervening mechanisms as well as boundary conditions of this effect [16]. The mediating mechanism will help us understand the underlying processes of psychological pathways, cognitive shifts, or emotional responses that connect the experience of ostracism to changes in IWB. In addition, the boundary conditions can help us understand how adverse impacts of workplace ostracism can be mitigated.

Thus, drawing on the conservation of resources (COR) theory, we propose a framework to investigate perceived control as an underlying mechanism between workplace ostracism and IWB. Furthermore, we endeavor to examine the moderating effects of paradox mindset and support for innovation. Perceived control refers to the influence one has in overcoming the adversity of painful situations. Research shows that individuals with high perceptions of control over their resources are more likely to alter their social environment for good [17]. Thus, when individuals encounter any unpleasant experience at the workplace, their perceptions of low control make them prone to lose motivation and the ability to influence their work and surroundings [18].

Furthermore, the paradox mindset promotes paradoxical cognitions, which help manage work stress [19]. Research has established that upon coming across potential stressors at the workplace, before applying any coping strategy or mechanism, individuals try to interpret and understand the experience to deal with it effectively [20]. Once employees are confronted with conflicting demands, organizational tensions are considered paradoxes, and a paradoxical mindset will try to respond innovatively [21]. It provides cognitive support to achieve positive outcomes in the face of workplace stressors [19]. Similarly, the support extended by the organization promotes eliciting innovative behavior from employees [22].

This study offers significant insights that deepen our understanding of workplace dynamics. Firstly, it addresses a notable research gap by investigating how workplace ostracism can discourage employees from engaging in constructive behaviors like IWB [23]. This fills a void in the current literature, where such examinations are limited. Secondly, this study responds to the need for research that uncovers the mechanisms between ostracism and IWB. Guided by the COR theory, our study bridges this gap by examining perceived control as a key resource. This study empirically analyzes how workplace ostracism influences IWB through the mediating link of perceived control. This sheds light on the underlying processes that shape the relationship between ostracism and IWB. Furthermore, this research attends to the call for understanding ways to mitigate the negative effects of ostracism, as suggested by Robinson, O’Reilly [24]. The study does this by systematically exploring the potential impact of two moderating mechanisms: the paradox mindset and support for innovation. These additional dimensions broaden our comprehension of how workplace ostracism interacts with different factors to influence IWB.

Finally, this study investigates these effects within the context of public sector employees in Pakistan. It is a well-established fact that creativity extends beyond traditional creative occupations [25, p. 356]. With this perspective in mind, we chose employees from public sector organizations as respondents. It is noteworthy that a substantial portion of workplace ostracism research has relied on private-sector employees as respondents. As far as the public sector is concerned, primarily educational institutions and hospitals’ employees have been studied, while other segments have been largely overlooked. There is a lack of comprehensive frameworks to address workplace ostracism for public sector employees. In light of the persistent criticism of low performance and inefficiency in service delivery within Pakistan’s public sector, this study stands to make a significant contribution. By examining the impact of workplace ostracism on IWB among public-sector employees, this research fills a crucial gap and offers insights that can aid in enhancing the performance of public-sector organizations.

In conclusion, the contributions of this study hold implications for both theory and practice. By addressing research gaps, unveiling mediating and moderating mechanisms, and elucidating the complex dynamics of ostracism’s impact on IWB, this research advances our knowledge in this area and offers valuable insights for organizations striving to foster innovation while navigating the challenges of interpersonal workplace dynamics.

## Theoretical foundations and hypotheses development

### The conservation of resources theory (COR)

The COR theory suggests that individuals need to conserve resources for their survival. Resources could be of personal, motivational, financial and social nature. Personal resources include physical health as well as psychological well-being [26]. When employees experience workplace ostracism, they undergo resource loss, which curtails their feelings of belongingness and affiliation [27]. The COR theory postulates that resource depletion occurring in response to stress leads to exhaustion and strain [27, 28]. Given that individuals possess limited physical, psychological, and emotional resources [29], by encountering ostracism, an individual’s limited resources deplete rapidly. Ostracism not only inhibits interactions among individuals but also obstructs meaningful collaborations within the workplace, ultimately impairing overall human functioning in that environment. The impact of ostracism extends from psychological well-being to practical behavioral functioning, showcasing its pervasive effects.

Hence, this study focuses on how ostracism erodes the individual resource of perceived control, subsequently undermining employees’ innovative behavior. Furthermore, it delves into the role of a paradox mindset, which constitutes another individual resource, in mitigating the adverse effects of ostracism. Support for innovation encompasses assistance provided by an organization to empower individuals in bolstering their personal resources, thereby fostering enhanced creative behaviors among employees [30].

### Workplace ostracism and innovative work behavior

Human beings are inherently reliant on others for their well-being. Individuals are wired in a manner that prevents them from realizing their full potential if they are deprived of meaningful connections with others. Such deprivation can have a direct impact on their physical and mental capabilities [31]. Thus, ostracism characterized by avoidance and rejection extended by either individuals or groups towards other employees and groups, can lead to distress [32]. It can be purposeful, where individuals are conscious of their actions, or non-purposeful, where individuals ignore others without intent, leading to hurt feelings and social pain [33].

In the workplace, innovation involves interactions with others as it is a socially embedded process where people establish interpersonal ties to enhance the acceptance of their ideas by others [34]. Ostracism inflicts distress and prompts maladaptive responses. It can lead individuals to participate less in social interactions and dampen their willingness to communicate and share information and ideas, potentially hindering individual innovative behavior [23, 35]. Research shows that the social environment significantly impacts creativity, either enhancing or inhibiting it [22]. Ostracism inhibits creativity as it causes ostracized individuals to miss out on crucial information and knowledge due to strained social relationships. Furthermore, it is associated with reduced voicing behavior and constrains employees from offering constructive suggestions [13, 24, 36].

Episodes of ostracism undermine employees’ work engagement [37] and overall well-being outcomes, including psychological well-being, emotions, and self-perception [38], thereby impeding innovative behavior. Thus:

***H1*.** *Workplace ostracism is negatively associated with innovative work behavior*.

### Workplace ostracism and perceived control

The COR theory posits that individuals require an ample supply of resources to perform human functions adequately [39]. Perceived control, a personal resource, reflects an individual’s belief in their ability to influence behaviors or situations, serving as a buffer against adversity [40]. Early theorists underscore the pursuit of control and autonomy as fundamental human endeavors, associated with elevated self-worth and hope when perceived, and conversely linked to low self-worth and hopelessness when absent. Moreover, low perceived control has been correlated with heightened symptoms of depression [41, 42].

Research has further suggested that engagement in self-regulatory or control activities depletes self-regulatory focus [43]. Experiencing ostracism instills a sense of control loss in individuals [3, 44, 45], as evidenced by experimental studies showing that socially excluded subjects exhibited lower self-regulation compared to their non-excluded counterparts [see, 44]. Perceived control functions as a psychological mechanism influencing individuals’ behaviors and internal states. Notably, individuals consistently self-evaluate to ensure a sense of control over their environment [29, 46]. Nevertheless, ostracism evokes feelings of insignificance and worthlessness, akin to a form of "social death" [47], notably undermining individuals’ perceptions of control. Based on the preceding discussion, we hypothesize:

***H2a*.** *Workplace ostracism is negatively associated with perceived control*.

### Perceived control and innovative work behavior

In line with the COR theory, exposure to stress-inducing events can erode employees’ self-evaluations, subsequently diminishing their perceptions of control [42]. According to the social cognitive theory, individuals endowed with higher levels of perceived control tend to excel in demanding and creative tasks, in contrast to their peers with lower control perceptions [48]. The COR theory also aligns with the basic psychological needs theory, which highlights autonomy, competence, and belongingness as pivotal psychological needs that function as essential resources, facilitating self-regulation, adaptation, and overall well-being [49]. Extensive research further establishes that the fulfillment of these fundamental needs fosters the emergence of IWB [50]. Research by Philippaers, De Cuyper [51] and Ucar, Hasta [52] confirms these findings, emphasizing the influence of perceived control on shaping behavioral intentions and its positive correlation with outcomes such as job performance and life satisfaction [29]. Furthermore, an empirical investigation by Zhang, Liu [53] affirms a positive association between perceived control behaviors and innovative intentions, wherein the latter directly contributes to driving creative behaviors.

Empirical evidence consistently indicates that individuals who harbor a sense of control demonstrate a heightened willingness to invest effort, engage in adaptive actions, and outperform their peers [29, 51]. Furthermore, the study conducted by Kennett, Quinn-Nilas [54] establishes a strong link between higher levels of perceived control and increased resilience, with individuals possessing a stronger sense of control also exhibiting greater resourcefulness. Given these compelling insights, we posit that perceived control functions as a potent precursor, significantly shaping employees’ inclination towards IWB within the framework of the COR theory. Thus, building upon this foundation, we propose the following hypothesis:

***H2b*.** *Perceived control is positively associated with innovative work behavior*.

### The mediating role of perceived control

In their extensive literature review, Skinner [55] suggested that the loss of control is a psychological trauma and is considered aversive. Notably, research by Kennett, Quinn-Nilas [54] illustrated the sequential mediation of learned resourcefulness and perceived control for the effect of academic stress on student resilience. In post-layoff scenarios perceived as more unsettling, Brockner, Spreitzer [56] found a stronger correlation between perceived control and performance. Vander Elst, De Cuyper [57] further indicated that personal control played a mediating role between job insecurity, a workplace stressor, and both physical and psychological well-being. Moreover, the significance of perceived control intensifies during times of stress, particularly when individuals perceive maltreatment, as evident in studies by Philippaers, De Cuyper [51] and Kennett, Quinn-Nilas [54]. Drawing on these insights, we advance the following hypothesis:

***H2c*.** *The negative association between workplace ostracism and innovative work behavior is mediated by perceived control*.

### The moderating role of paradox mindset

Mindset-based interventions are increasingly gaining importance in contemporary times [58], influencing how individuals perceive and engage with their work responsibilities [59, 60]. In recent years, researchers have focused on understanding the dynamics of tensions and their outcomes within organizations [20, 61]. Tensions often arise due to limited resources in the workplace, and one’s mindset plays a crucial role in promoting positive outcomes, including IWB, psychological well-being, resilience, and leadership effectiveness [59]. The concept of a growth mindset is particularly noteworthy, as it serves as a personal asset that fosters engagement by guiding attention, enthusiasm, and interpersonal interactions [62, 63].

Central to our study is the notion of paradox mindset, which involves an individual’s cognitive approach to embracing contradictions and conflicts as challenges. Those who possess a paradox mindset tend to be more comfortable with tensions [20, 21], contributing to optimism [64], work engagement, and proactive behaviors [65]. Moreover, paradox mindset enhances cognitive flexibility, enabling individuals to tolerate differences [66, 67]. In the context of workplace ostracism, paradox mindset interprets it as a type of organizational tension, prompting the generation of innovative ideas and strategies to address it. Additionally, during periods of stress, the paradox mindset aids in conserving resources to effectively carry out job tasks. Drawing from the aforementioned discourse, we propose the following hypothesis:

***H3*.** *Paradox mindset moderates the negative association of workplace ostracism and perceived control in such a way that this effect is low with a high paradox mindset*, *and the effect is high with a low paradox mindset*.

### The moderating role of support for innovation

Numerous contextual factors play a significant role in shaping IWB. Notable among these factors are leadership styles, organizational attributes, and broader national culture, all of which exert considerable influence on employees’ inclination towards innovative behavior [68]. The support for innovation encompasses both the climate for innovation and enacted support. Climate for innovation is discernible through policy documents and informal communication, while enacted support involves active facilitation of innovative behaviors [69]. An investigation by Hsiao, Chang [69] revealed that the extent of an organization’s support for innovation profoundly impacts individual behaviors. Amabile and Conti [23] asserted that employees within organizations that nurture creativity, recognize, and reward innovative tasks are more likely to exhibit creative behaviors [70].

Moreover, the ongoing growth of support for innovation underpins continuous innovation, underscoring its paramount importance in today’s environment [71]. Gumusluoğlu and Ilsev [72] posited that organizational values, culture, and norms are potential influencers of employees’ creative capacity. Recent research by Afshar Jahanshahi, Adiguzel [73] also highlighted that the innovation management process is contingent upon organizational values and belief systems. Additionally, a study conducted by Demircioglu [74] underscored that an innovation-supportive environment can enhance commitment and engagement among employees, ultimately contributing to job satisfaction. In light of the above, we propose the following hypothesis:

***H4*.** *Support for innovation moderates the positive association of perceived control and innovative work behavior in such a way that this relationship will be more pronounced when support for innovation is high rather than when it is low*.

Based on the above, we formulate the moderated mediation hypothesis as follows:

***H5*.** *The negative effect of ostracism on innovative work behavior through perceived control would be buffered when paradox mindset and support for innovation are high*.

**Fig 1:**
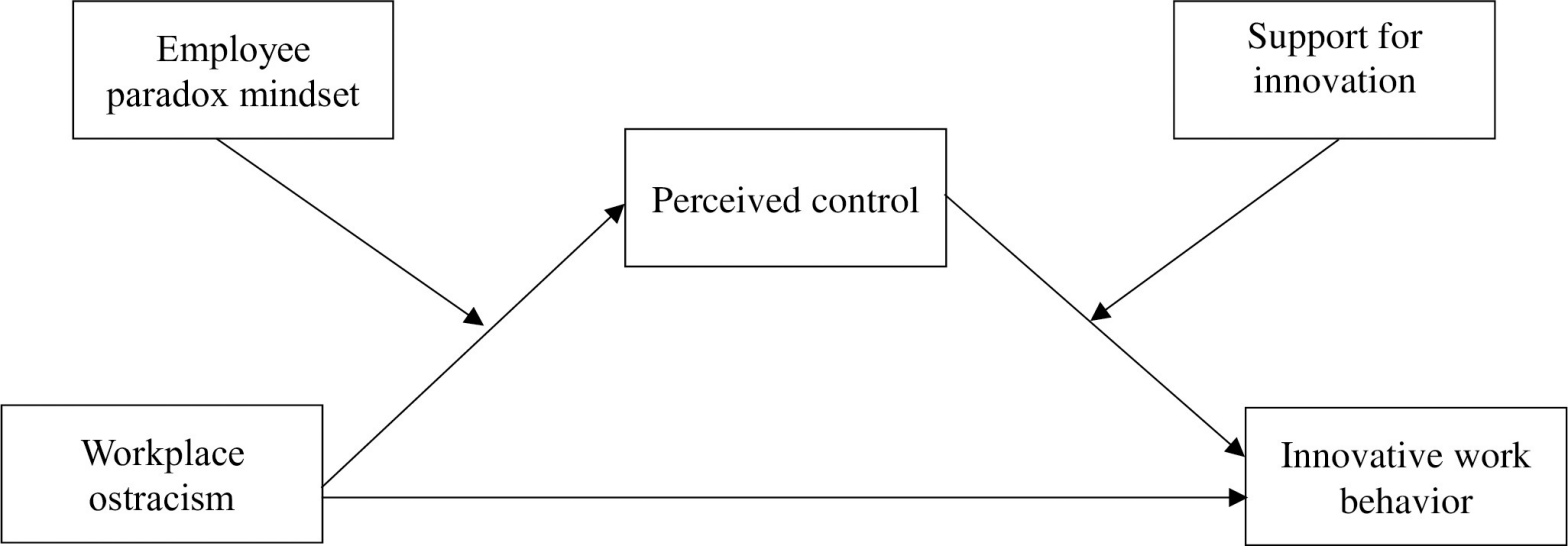
Proposed relationships of workplace ostracism and innovative work behavior.

The conceptual framework is presented in Fig 1.

## Methods

### Participants and procedure

This study employed a time-lagged quantitative approach, with data collected at three intervals. The sample consisted of 513 public sector employees of Pakistan. The reason behind using quantitative study design in this study is that it has many potential benefits, that is, it is relatively easy, quick, less expensive and provides more understandable and useful results. Moreover, it is easier to form generalizations and replicability from the results of the quantitative study. Closed-ended questionnaires were used for data collection in this study because it helps to collect responses from a large number of respondents and make coding easy for analysis [75].

Given the central focus on addressing perceived workplace ostracism among individuals, the unit of analysis was individual employees working in departments such as local government, community development, planning and development, services and general administration, labor, and human resource departments. With inefficiency being prevalent in the public sector and human resources playing a pivotal role in organizational efficiency, the public sector provided an appropriate context to understand how the impact of workplace stressors on individual IWB could be mitigated.

Approval from the "Institutional Review Board" of Government College University Faisalabad, Pakistan, was obtained. Further, respondents were informed that their responses would be kept confidential and that their participation was voluntary. We obtained written informed consent from the respondents when questionnaires were got filled. Questionnaires were distributed by visiting offices in person as well as through online mediums. Surveys were administered in the English language, as English is the official language of communication in government offices of Pakistan.

A time-lagged research design was employed, consisting of three waves of data collection, with a 4-week interval wedged between each wave. It serves to curtail the common method bias (CMB) issue, and the causal mediation analyses inherent in our study render the time interval wedged between waves of data collection meaningful, as time is required for effects to manifest [76]. The data for workplace ostracism and paradox mindset was obtained at T1; at T2, the respondents rated perceived control and support for innovation; whereas at T3, IWB ratings were obtained. Seven hundred questionnaires were distributed at T1, yielding 660 responses, of which 30 questionnaires were discarded due to incomplete responses. In the subsequent T2 wave, occurring a month later, the 630 remaining respondents were contacted through email/WhatsApp and office visits, resulting in 580 responses, 25 of which were incomplete and therefore discarded. The researchers contacted the same 555 respondents at T3 wave, yielding 520 responses. Out of these 520 responses, 7 responses were discarded due to incomplete data, resulting in *n* = 513 with a response rate of 73.3%.

### Measures and variables

Responses for all the items were collected on a 5-point Likert scale ranging from "1 = strongly disagree to 5 = strongly agree."

Workplace ostracism was evaluated using a 10-item questionnaire developed by Ferris, Brown [6]. A sample item is "others ignored me at work." We measured perceived control using a 3-item instrument from Lee, Ashford [28]. A sample item is "I can prevent negative things from affecting my work situation." IWB was measured using a 10-item measure from De Jong and Den Hartog [77]; a sample item includes "I find new approaches to execute tasks." A 9-item questionnaire from Miron-Spektor, Ingram [19] was used to measure paradox mindset; a sample item out of this scale is "when I consider conflicting perspectives, I gain a better understanding of an issue." Support for innovation was measured by using 5 items developed by Siegel and Kaemmerer [78] & Scott and Bruce [79], also used by Choi, Moon [80]. A sample item is "Creativity is encouraged here."

### Control variables

In our analysis, we accounted for several demographic characteristics of respondents as control variables, including gender, age, education, and experience. We included gender as a control variable due to findings from previous studies indicating that, when subjected to ostracism, females tend to experience more pronounced harmful effects and psychological distress than males [81]. Similarly, Rajchert, Konopka [82] highlighted the significant impact of gender differences on individuals’ post-ostracism behavior. Additionally, age, education, and experience were controlled for, as these factors commonly influence employee organizational behaviors [44]. Notably, age was found to exhibit a negative correlation with ostracism, implying that adults report experiencing ostracism less frequently [83]. Madrid, Patterson [34] stated that when employees have more work experience, it gives them confidence in innovative ideas. Carmeli and Spreitzer [84] prompted us to control education level and age, as they hypothesized that these variables might yield distinct positive or negative effects on innovation.

## Results

### Demographics

The demographic profile of the respondents is given in Table 1.

**Table 1:** Sample characteristics.

	*f*	*%*
Gender		
Female	173	33.7%
Male	340	66.3%
Age		
Less than 30 years	189	36.8%
31–40 years	322	62.8%
41–50 years	2	0.4%
Education		
Masters	244	47.6%
MPhil/MS	269	52.4%
Work experience		
Less than 5 years	240	46.8%
5–10 years	217	42.3%
11–15 years	56	10.9%
Job level		
BPS-16	92	17.9%
BPS-17	338	65.9%
BPS-18	83	16.2%

### Confirmatory factor analysis (CFA)

Most constructs had a sizeable number of items, so the item parceling technique was applied to analyze the final model [85] for CFA. This study used a random algorithm for item parceling, randomly assigning items to a parcel [85]. The items of workplace ostracism, IWB, and paradox mindset, thus, were categorized into three parcels each. Perceived control had three items, and support for innovation had five items. Hence, these variables were measured by their specific individual items.

The results of CFA revealed that the measurement model showed an excellent fit, as the goodness-of-fit indices were comfortably above the acceptable thresholds (see Table 3, Model-1). Furthermore, Table 2 presents that factor loadings are higher than the benchmark value of 0.70, the construct values for composite reliability (CR) and Cronbach’s α are higher than the criterion value of 0.70, and the construct values of average variance extracted (AVE) are above the criterion value of 0.50. Therefore, the outcomes confirm convergent validity.

**Table 2:** Factor loadings, Cronbach’s Alpha (α), composite reliability and AVE.

Variables	Loading	Cronbach’s α	Composite Reliability	AVE
Ostracism				
Ostracism parcel 1	0.84**	0.85	0.79	0.56
Ostracism parcel 2	0.68**			
Ostracism parcel 3	0.71**			
Perceived control				
Perceived control parcel 1	0.80**	0.71	0.74	0.50
Perceived control parcel 2	0.80**			
Perceived control parcel 3	0.47**			
Innovative work behavior				
IWB parcel 1	0.85**	0.83	0.75	0.52
IWB parcel 2	0.79**			
IWB parcel 3	0.46**			
Paradox mindset				
Paradox mindset parcel 1	0.53**	0.83	0.77	0.53
Paradox mindset parcel 2	0.82**			
Paradox mindset parcel 3	0.80**			
Support for innovation				
Support for innovation 1	0.58**	0.94	0.94	0.78
Support for innovation 2	0.89**			
Support for innovation 3	0.96**			
Support for innovation 4	0.97**			
Support for innovation 5	0.95**			

*Note(s)*. AVE = Average variance extracted

**p < 0.01

The discriminant validity was evaluated by analyzing the initial five-factor model in comparison with other possible measurement models, as given in Table 3, which affirmed the superiority of the proposed five-factor model’s fit over others. In addition, the Fornell and Larcker [86] criterion presented that the square root of AVE of all variables is higher than that particular variable correlations with others, as given in Table 4. These outcomes confirm discriminant validity.

**Table 3:** Comparison of alternative measurement models for main constructs.

	Model	Factors	χ^2^(df)	χ^2^/df	RMSEA	SRMR	GFI	NFI	TLI	CFI	Model Comparison	Δχ^2^	Δdf
1	Hypothesized five-factor model	Ostracism; perceived control; innovative work behavior; paradox mindset; support for innovation	206.05**(109)	1.89	.042	.0422	.96	.96	.98	.98	-	-	
2	Four-factor model	Ostracism; perceived control; innovative work behavior + support for innovation; paradox mindset	992.99**(113)	8.79	.123	.1669	.80	.82	.81	.84	2 versus 1	786.94**	4
3	Two-factor model	Ostracism + perceived control + paradox mindset; innovative work behavior + support for innovation	1468.41**(118)	12.44	.150	.1761	.72	.73	.71	.75	3 versus 1	1262.36**	9

**p < .01

**Table 4:** Descriptive statistics and observed correlations.

		Mean	SD	1	2	3	4	5	6	7	8	9	10
1	Gender	.66	.47	-									
2	Age	1.64	.49	-.03	-								
3	Education	2.52	.50	.01	-.15**	-							
4	Experience	1.64	.67	-.07	.45**	-.04	-						
5	Job level	1.98	.58	.00	.39**	-.22**	.44**	-					
6	Ostracism	2.61	.88	.10*	.02	.04	.09*	-.04	*(0*.*75)*				
7	Perceived control	3.97	.81	-.06	.01	.03	-.03	-.01	-.31**	*(0*.*70)*			
8	Innovative work behavior	3.58	.76	-.05	.00	-.05	-.05	.03	-.65**	.36**	*(0*.*72)*		
9	Paradox mindset	3.60	.69	-.03	.01	.02	-.06	.05	-.43**	.37**	.50**	*(0*.*73)*	
10	Support for innovation	3.52	1.10	.07	.01	.06	-.03	.02	-.04	.09*	.07	.12**	*(0*.*88)*

*Note*. N = 513. The diagonal elements showcase the square root of AVE; while the estimated correlations are depicted below the diagonals.

*p < .05

**p < .01

### Common method bias (CMB)

To overcome the issue of CMB, different procedural remedies were applied. The respondents were instructed that they should remain honest while answering the questions. To avoid social desirability bias, the identities of the individuals were kept anonymous [87]. Moreover, to avoid CMB, a time-lagged study design was used. By wedging temporal separation in the form of time lags, the respondents could be restricted from using familiar cues to retrieve information from long-term memory [87].

To estimate the common variance, we used the common latent factor (CLF) method following Podsakoff, MacKenzie [88]. We resorted to using CLF as the previously widely used Harman’s single-factor test is considered outdated and an approach with certain limitations [89]. In the CLF method, a new latent variable is introduced, called CLF, which is linked directly to all the observed variables. We constrained all the factor loadings from CLF to the observed variables as equal, and the variance of the common factor was constrained to 1. The estimate of common variance can be calculated by taking the square of the equally constrained loading of the common factor to the observed variables before standardization. The common variance for our model was estimated to be 0.04 or 4% by computing the square of 0.206 (equally constrained loading of CLF to the observed variables). This common variance is much lower than the proposed threshold of 50% [90], thus indicating that the presence of CMB can be safely ruled out.

### Descriptive statistics and correlations

As can be evidenced from Table 4, ostracism was found to have a negative correlation with perceived control (-.31, p < .01) and IWB (-.65, p < .01). Perceived control had a positive correlation with IWB (.36, p < .01). Paradox mindset had a positive correlation with perceived control (.37, p < .01), while support for innovation’s correlation was insignificant with IWB (.05, p > .05).

### Hypotheses testing

Regression results from PROCESS macro model 4 (see Table 5) showed that ostracism showed a negative relationship with perceived control (-.28, p < .01) as well as with IWB (-.52, p < .01). Thus, H1 and H2a were supported. Perceived control had a positive effect on IWB (.16, p < .01); therefore, H2b was supported.

**Table 5:** Parameter estimates.

Independent Variable	Outcome	
	*M*: Perceived control	*Y*: Innovative work behavior
Main effects		
*X*: Ostracism	-.28**(.04)	-.52**(.03)
*M*: Perceived control	-	.16** (.03)
Control variables		
Gender	-	.04
Age	-	.02
Education	-	-.05
Experience	-	.00
Job level	-	.00

*Note*. N = 513. Values in parentheses show standard error of the respective parameter estimate

*p < .05

**p < .01

### Mediation analysis

Results reported in Table 6 support H2c regarding the mediation of perceived control. Employing bootstrapping procedure supplied in the PROCESS macro, the results reported in Table 6 revealed that perceived control significantly mediated the relationship between ostracism and IWB (indirect effect = -0.05, p < 0.01). Therefore, H2c was supported.

**Table 6:** Total, direct and indirect effects of ostracism on innovative work behavior.

Effect	Product of Coefficients	SE	BC 95% CI^1^	
			Lower	Upper
*Indirect effect*	ab			
Ostracism—> Perceived control—> Innovative work behavior	-.28*.16 = -.05**	.01	-.08	-.02
*Direct effect*				
Direct effect (c’)	-.52**	.03	-.62	-.51
*Total effect*				
Total effect (c)	-.56**	.03	-.58	-.46
*R-Square*				
Perceived control				.10
Innovative work behavior				.45

*Note*. N = 513

**p < .01

^1^ This 95% confidence interval does not include zero; therefore, the mediating effect is significant at p < .05

### Moderated mediation analysis

The PROCESS macro model 21 was used to evaluate moderated mediation. The results in Table 7 reveal that perceived control was significantly affected by the interaction of ostracism and paradox mindset (ostracism*paradox mindset = 0.33, p < .01). Likewise, IWB was significantly predicted by the interaction of perceived control and support for innovation (perceived control*support for innovation = 0.06, p < .05). These findings indicated that paradox mindset and support for innovation had a moderating effect on the link between ostracism and perceived control and the relationship between perceived control and IWB, respectively. Thus, H3 and H4 were supported.

**Table 7:** Results of the moderated mediation analysis.

	*M*: Perceived control			*Y*: Innovative work behavior		
	B	SE	95% CI	B	SE	95% CI
*X*: Ostracism	-1.26**	0.17	-1.59, -.93	-0.52**	0.03	-.58, -.46
*W*: Paradox mindset	-0.60**	0.15	-.89, -.30			
Ostracism*Paradox mindset	0.33**	0.05	.23, .43			
*M*: Perceived control				-0.05	0.10	-.25, .15
*V*: Support for innovation				-0.21	0.11	-.43, .01
Perceived control*Support for innovation				0.06*	0.03	.01, .11
Constant	6.40	0.54	5.33, 7.47	5.04	0.42	4.21, 5.86
	R^2^ = .23			R^2^ = .46		
	F = 51.51** (3, 509)			F = 107.01** (4, 508)		

*Note*. SE = Standard error, CI = Confidence interval

**p < .01

*p < .05

The moderation analysis results reveal that the negative impact of ostracism on perceived control is neutralized if the employees have paradoxical mindset. It can be seen from the conditional effects reported in Table 8 that as the score of the paradox mindset increases, the effect of ostracism on perceived control becomes less negative. This indicates alleviation of the negative effect of ostracism on perceived control as the score for the paradox mindset increases. In addition, the positive effect of perceived control on IWB is enhanced in case the organization offers support for innovation. Conditional effects depict a strengthening of the positive impact of perceived control on IWB as the score for support for innovation increases (see Table 8).

**Table 8:** Conditional effects of the focal predictor at values of the moderator.

Focal predictor: Ostracism						
Moderating variable: Paradox mindset						
Outcome variable: Perceived control						
Paradox mindset	Effect	SE	t	p	LLCI	ULCI
Mean - 1SD	-0.30	0.04	-6.77	0.00	-0.39	-0.21
Mean	-0.07	0.04	-1.71	0.09	-0.15	0.01
Mean + 1SD	0.16	0.06	2.49	0.01	0.03	0.28
Focal predictor: Perceived control						
Moderating variable: Support for innovation						
Outcome variable: Innovative work behavior						
Support for innovation	Effect	SE	t	p	LLCI	ULCI
Mean - 1SD	0.10	0.04	2.19	0.03	0.01	0.18
Mean	0.16	0.03	4.95	0.00	0.10	0.23
Mean + 1SD	0.23	0.04	5.06	0.00	0.14	0.31

SE = standard error, LLCI = lower-level confidence interval, ULCI = upper level confidence interval

Combined with the whole model, the results revealed that the negative effect of ostracism on IWB through perceived control would be alleviated effectively when the scores of paradox mindset and support for innovation were high. Conditional indirect effects reported in Table 9 reveal that the negative indirect effect of ostracism on IWB through the mediation of perceived control would gradually become less negative as the score for paradox mindset increases for low, average, and high values of the support for innovation. As shown in Table 9, the index of moderated mediation was significant (Index = 0.02, CI = 0.002–0.038). H5, therefore, was supported.

**Table 9:** Conditional indirect effects of X on Y.

Indirect effect					
Ostracism -> Perceived control -> Innovative work behavior					
Paradox mindset	Support for innovation	Effect	BootSE	BootLLCI	BootULCI
Paradox mindset Mean - 1SD	Support for innovation Mean - 1SD	-0.03	0.02	-0.063	-0.001
	Support for innovation Mean	-0.05	0.01	-0.080	-0.022
	Support for innovation Mean + 1SD	-0.07	0.02	-0.106	-0.033
Paradox mindset Mean	Support for innovation Mean - 1SD	-0.01	0.01	-0.022	0.001
	Support for innovation Mean	-0.01	0.01	-0.028	0.001
	Support for innovation Mean + 1SD	-0.02	0.01	-0.037	0.002
Paradox mindset Mean + 1SD	Support for innovation Mean - 1SD	0.01	0.01	0.000	0.035
	Support for innovation Mean	0.03	0.01	0.006	0.049
	Support for innovation Mean + 1SD	0.04	0.02	0.008	0.068
Index of moderated mediation					
		Index	BootSE	BootLLCI	BootULCI
		0.02	0.01	0.002	0.038
Indices of conditional moderated mediation by Paradox mindset					
	Support for innovation average	Index	BootSE	BootLLCI	BootULCI
	2.41	0.03	0.02	0.001	0.066
	3.52	0.05	0.02	0.025	0.086
	4.62	0.07	0.02	0.037	0.116

BootSE = Bootstrap standard error, BootLLCI = Bootstrap lower-level confidence interval, BootULCI = Bootstrap upper level confidence interval

## Discussion

The objective of this study was to examine the intricate connection between workplace ostracism and IWB via perceived control, along with investigating the moderating effects of paradox mindset and support for innovation. The impact of workplace ostracism has debilitating influence on social and emotional resources due to limited interactions. As work is significantly dependent on idea exchange and interactions with colleagues, ostracism-induced hindrance of these interactions impairs knowledge sharing and support, directly affecting IWB, thus validating H1.

Hypothesis H2a posited a negative link between workplace ostracism and perceived control, a relationship that has been substantiated by our findings. Consistent with the COR theory, which asserts that individuals endeavor to safeguard and augment their resources, our study confirms that workplace ostracism, as a stress-inducing factor, results in the depletion of resources. This depletion subsequently diminishes individuals’ perceptions of control over their environment. Previous research has already illuminated the detrimental effects of ostracism on employees’ social and psychological well-being, including burnout [7] and emotional exhaustion [8]. Zadro, Williams [91] extended these insights by demonstrating that ostracized participants reported diminished levels in the measurement of their four fundamental psychological needs. However, the connection between ostracism and perceived control remains relatively underexplored. Our study contributes to the existing body of literature by providing empirical support for Hypothesis H2a, thus enriching our understanding of the intricate dynamics between workplace ostracism and individuals’ perceived control.

Hypothesis H2b proposed that perceived control is positively linked with IWB. The social cognitive theory offers support to the claim that individuals with higher cognitive abilities tend to excel in creative and challenging tasks [48]. Control perceptions, identified as fundamental psychological needs, enhance cognitive functioning, reduce stress, and thereby contribute to superior performance in innovative tasks [51, 53]. Our study verifies this hypothesis, emphasizing that employees who possess a stronger sense of personal control are more inclined to embrace challenges and demonstrate a higher likelihood of engaging in IWB. Furthermore, Hypothesis H2c posited that the negative association between workplace ostracism and IWB is mediated by perceived control, and our findings provided support for this mediation effect. Our research uncovers a significant insight–that the impact of workplace ostracism on IWB is, in part, channeled through perceived control. This suggests that enhancing perceived control could be a crucial strategy for mitigating the adverse effects of workplace ostracism on employees’ propensity for IWB.

Our study substantiates H3, which proposed the moderating role of the paradox mindset in the context of workplace ostracism and perceived control. The findings reveal that, under the boundary condition of individuals possessing paradox mindset, the adversity posed by ostracism can be mitigated. Specifically, our study found that employees characterized by lower levels of paradox mindset were more susceptible to the detrimental impact of workplace ostracism. However, for those exhibiting elevated levels of paradox mindset, the otherwise negative and adverse effects of ostracism appeared to be less pronounced and more manageable.

This moderation effect aligns with insights drawn from Miron-Spektor, Ingram [19], as well as from Smith and Lewis [61], who propose that the paradox mindset equips individuals with effective tools for managing tensions and contradictions. Our empirical findings provide robust support for the notion that the paradox mindset serves as a protective mechanism, offsetting the adverse consequences of workplace ostracism. By validating the moderating influence of paradox mindset on the association between workplace ostracism and perceived control, our study contributes to the expanding domain of mindset interventions. Notably, this goes beyond its conventional role as a coping strategy for workplace ostracism, showcasing the paradox mindset’s potential of transforming adversity into positive outcomes. In essence, our research enhances our understanding of the intricate interplay between psychological factors and workplace dynamics, providing valuable insights for interventions aimed at fostering resilience and improved well-being among employees.

Our findings validate H4, highlighting the role of support for innovation in enhancing the connection between perceived control and IWB. This resonates with the previous research [see, for instance, 69,73], indicating that organizations promoting employee efforts tend to foster a creative workplace environment. Moreover, our study underscores the reinforcing influence of support for innovation on the positive relationship between perceived control and IWB. This aligns with Van de Ven [92] on innovation as a collective process, emphasizing the significance of organizational support and top management cooperation.

H5 posited that the adverse influence of ostracism on IWB, mediated by perceived control, would be alleviated under high levels of both paradox mindset and support for innovation. This extension of boundary conditions finds coherence within our study’s theoretical framework. By considering the combined effects of paradox mindset and organizational innovation support, our research illuminates a multifaceted approach to buffering the negative consequences of ostracism on IWB. The interplay between these factors contributes to a comprehensive understanding of how individuals navigate challenges and continue to engage in creative initiatives despite adversities.

### Theoretical implications

This study substantiates our hypothesized research model, employing perceived control as a personal resource to mitigate the stress induced by ostracism within the boundary conditions of paradox mindset and support for innovation. Notably, our findings have noteworthy implications for theoretical advancements. A significant contribution lies in the introduction of perceived control as a proximal antecedent of IWB, elucidating the linkage between workplace ostracism and IWB. By establishing perceived control as a mediating mechanism, this study bridges gaps within ostracism and innovation literature, shedding light on the ostracism-IWB nexus. Consequently, our research enriches IWB literature by introducing a robust predictor.

Furthermore, our exploration extends the paradox mindset literature by employing it as a boundary condition for the association between ostracism and perceived control. Unveiling the paradox mindset as a moderator provides valuable insights into the nuances of cognitive resource depletion and effective coping strategies among ostracized individuals. This elucidates when and why employees perceive ostracism as a challenge and how they effectively navigate it. Additionally, our study contributes to the discourse on support for innovation as a moderator for the perceived control-IWB relationship. The significant role of support for innovation in reinforcing the positive connection between perceived control and IWB is underscored, adding depth to our understanding.

Moreover, our research advances the field by empirically examining the impact of ostracism in real-time workplace settings, in contrast to the prevalent experimental manipulations in laboratory settings. Our study stands as one of the few conducted in real-time, offering a valuable perspective on workplace ostracism’s effects and coping mechanisms. In conclusion, our study’s multifaceted theoretical contributions enhance our comprehension of the intricate interplay between psychological factors, organizational dynamics, and individual behavior.

## Practical implications

By establishing perceived control as a robust intervening mechanism, the findings of this research equip managers with tools to boost employees’ personal resources. While the complete elimination of workplace stress may be impractical, its mitigation can be achieved through the strengthening of personal resources. Consequently, this research guides managers towards a focus on effective people management. Healthy workplace associations foster meaningful communication, facilitating access to knowledge resources and career advancement. Recognizing the value of paradox mindset in fostering coexistence with tensions and driving optimal performance [64], nurturing this mindset becomes pivotal. Managers, as influential agents, can significantly shape employees’ feelings, thoughts, and actions. Hence, it becomes imperative for managers to facilitate the development of paradoxical mindset. Providing support and constructive feedback when employees confront challenges or organizational tensions can effectively cultivate this mindset within employees.

Furthermore, a key implication of this study is that innovative behavior in the workplace is not solely an organizational interest but equally an individual pursuit [35]. Individuals with heightened control perceptions should be underpinned by organizational practices and policies geared towards fostering innovation. Organizational frameworks should be thoughtfully designed to encourage and support employees exhibiting innovative potential. In conclusion, our research underscores the need for managers to harness the power of perceived control, promote a paradox mindset, and align organizational practices with individual innovation interests. By adopting these insights, organizations can create environments conducive to enhanced well-being, creativity, and thriving.

## Limitations and future research directions

While this study possesses notable strengths, several limitations warrant consideration. The response rate and sample size were constrained by time limitations, potentially affecting the generalizability of results. Future research could mitigate this by employing larger and more diverse samples to enhance result applicability. Moreover, our study predominantly focused on the individual level, providing a narrower perspective within a broader context. Future investigations may adopt multilevel and multisource methodologies to provide a more comprehensive understanding of workplace ostracism, its underlying mechanisms, and boundary conditions. Variations across public and private entities could yield more nuanced insights and generalize findings more effectively.

Despite utilizing a time-lagged design with inherent advantages over cross-sectional methods, future studies employing longitudinal designs could yield richer insights into workplace ostracism’s effects over extended periods. Cultural factors can exert an influence on study outcomes. Given this study’s focus on Pakistan, cross-cultural studies in different settings could unveil culture-specific outcomes. Furthermore, supplementing close-ended questionnaires with qualitative data collection could enhance the nuanced comprehension of employees’ perceptions of workplace ostracism.

Furthermore, the substantial role of the paradox mindset in mitigating the adverse effects of workplace ostracism suggests promising avenues for further exploration. Future research could delve into practical implementations of mindset interventions within employee contexts. In conclusion, while acknowledging these limitations, this study provides a foundational platform for future research endeavors. This future work holds the potential to expand and refine our understanding of workplace ostracism, its multifaceted consequences, and the potential interventions that could lead to healthier workplace dynamics and employee well-being.

## Conclusion

In conclusion, this study sheds light on the complex interplay between workplace ostracism, perceived control, and IWB. By exploring the roles of paradox mindset and support for innovation, this research provides valuable insights into how individuals respond to workplace challenges. The findings contribute to a deeper understanding of the dynamics that influence employees’ ability to innovate and thrive in the face of adversity, offering practical implications for organizations aiming to cultivate a positive and innovative work environment. As workplaces evolve, this study’s insights provide a valuable foundation for enhancing employee well-being and promoting creativity within organizations.

## Supporting information

S1 Data (RAR)
